# A Multilevel Analysis of Real-World Variations in Oral Anticoagulation Initiation for Atrial Fibrillation in Valencia, a European Region

**DOI:** 10.3389/fphar.2017.00576

**Published:** 2017-08-24

**Authors:** Aníbal García-Sempere, Daniel Bejarano-Quisoboni, Julián Librero, Clara L. Rodríguez-Bernal, Salvador Peiró, Gabriel Sanfélix-Gimeno

**Affiliations:** ^1^Center for Public Health Research (CSISP-FISABIO) Valencia, Spain; ^2^Spanish Network of Chronic Care and Health Services Research (REDISSEC) Valencia, Spain; ^3^Navarrabiomed Biomedical Research Centre Pamplona, Spain

**Keywords:** analysis of variance, anticoagulants, atrial fibrillation, drug utilization, multilevel analysis

## Abstract

**Introduction:** Beyond clinical trials, clinical practice guidelines, and administrative regulation, treatment decision-making can be influenced by individual and contextual factors. Our goal was to describe variations in the patterns of initiation of anticoagulation therapy in patients with atrial fibrillation by Health Areas (HA) in the region of Valencia in Spain and to quantify the influence of the HAs on variations in treatment choice.

**Methods:** We conducted a population-based retrospective cohort study of all atrial fibrillation patients who started treatment with oral anticoagulants between November 2011 and February 2014 in each of the region's 24 HAs. We described patient and utilization characteristics per HA and initiation patterns over time, and we identified contextual and individual factors associated with differences in initiation patterns.

**Results:** 21,879 patients initiated treatment with an oral anticoagulant in the 24 HAs. Initiation with direct oral anticoagulants (DOAC) in the first year was 14.6%. In November 2013 the ratio was 25.4%, with HA ratios ranging from 3.8 to 57.1%. DOAC-initiating patients had less comorbidity but were more likely to present episodes of previous ischemic stroke, hemorrhagic stroke, or TIA when compared with patients initiating with VKA treatment. Variability among HAs was statistically significant, with the majority of HAs ranking above or below the regional initiation average (ICC ≈ 8%).

**Conclusion:** There was high variability in the percentage of DOAC initiation and in the choice of DOAC among HAs. Interventions aimed to improve DOAC initiation decision-making and to reduce variations should take into account the Health Area component.

## Introduction

Atrial fibrillation (AF) increases the risk of stroke, which in turn, leads to cerebrovascular morbidity, neurological disability, loss of quality of life, and death (Jørgensen et al., 1996; Lin et al., 1996). Population-based studies in developed countries have shown a strong age gradient, with a prevalence of 6.6 men and 3.9 women for every 1,000 people of the respective gender (Chugh et al., 2014). Vitamin K antagonists (VKA) such as warfarin are highly effective in the prevention of stroke in AF patients, with several randomized clinical trials showing decreases in its incidence of more than 50% (1991; 1994; Petersen et al., 1989; The Boston Area Anticoagulation Trial for Atrial Fibrillation Investigators et al., 1990; Lancaster et al., 1991). Consequently, the use of VKA has been the standard therapy for AF patients at the highest risk of a stroke (Fuster et al., 2012; January et al., 2014). However, the treatment withVKA is subject to many limitations such as a higher risk of bleeding, the requirement of regular monitoring, and the presence of several drug–drug and drug–food interactions.

In recent years, novel direct oral anticoagulants (DOACs) such as dabigatran, rivaroxaban, apixaban, and edoxaban, have entered the market following the pivotal phase-III trials reporting efficacy and safety features and rates comparable to those of warfarin (Connolly et al., 2009; Granger et al., 2011; Patel et al., 2011; Giugliano et al., 2013). DOACs are considered a more convenient therapy, as they have lesser interactions than VKA treatments, simpler dosing regimes and the absence of need for INR monitoring. However, they also have important limitations, such as the low availability of antidotes to reverse their effect, their renal implications, the current lack of tests for monitoring their anticoagulant effectiveness, and the fact that the cost per day of treatment with DOACs is much higher than that of VKAs, including monitoring costs. Recently, real-world studies are shedding some light on whether or not the advantages of DOACs demonstrated in pivotal trials are being reflected in routine clinical practice (Potpara and Lip, 2017). Closely linked to safety and effectiveness outcomes in daily practice, the study of local patterns of use can provide relevant information for improving the management of patients with atrial fibrillation, and is essential to adequately interpret local real-world evidence.

To the best of our knowledge, patterns of real-world use of VKA and DOAC have barely been studied in other contexts (Brais et al., 2015; Olesen et al., 2015; Patel et al., 2015). In Spain, only one recently published study tackles this issue (Rodríguez-Bernal et al., 2017). DOAC prescription is subject to prior authorization in the Spanish National Health System, compelling the prescriber to get its prescription validated—by a so-called “medical inspector”—before it is accepted for public funding and dispensing. This validation relies on criteria fixed by the Ministry of Health. These criteria place DOAC as a second *line* therapy, and initiation of treatment with a DOAC is restricted to VKA contraindication, impossibility of accessing INR control facilities, or being at high risk of intracranial hemorrhage (ACOD, 2016). According to these formal strict regulatory rules, little variations in patterns of use, and initiation with DOAC among territories and neighboring populations with very similar demographic and epidemiological characteristics should be expected.

However, beyond the evidence provided by clinical trials, the recommendations of clinical practice guidelines, and the rules of administrative regulation, treatment decisions and the choice of a particular drug can be influenced by patient characteristics, physician, and organizational factors, pharmaceutical promotion, and healthcare system characteristics (Eisenberg, 2002). While a significant amount of literature about small area variations analysis (SAVA) in surgical and medical hospitalizations and healthcare spending has been an essential element for interpreting the behavior of healthcare providers and defining public policies (Wennberg et al., 2002; Fisher et al., 2003a,b; National Research Council., 2013), studies examining geographical variations in pharmaceutical prescribing have been scarce (Anis et al., 1996; Dubois et al., 2002) until recent years (Zhang et al., 2010a,b, 2012; Donohue et al., 2012) and, to our knowledge, none have addressed the contextual variability in VKA and DOAC drug utilization. Furthermore, because stroke prevention in atrial fibrillation treatment can be managed by several medical specialists (hematologists, cardiologists, and neurologists, among others) and primary care physicians, the role of the different levels of care explaining the geographical variations seems of special interest. This study aims to describe variations in the patterns of initiation of anticoagulation therapy in patients with atrial fibrillation in the region of Valencia in Spain, and to quantify the influence of the geographical healthcare administrative boundaries (Health Areas) on variations in treatment choice.

## Methods

### Design and setting

This population-based retrospective cohort study was conducted in the Valencia Health Agency (VHA), the public health system of the region of Valencia in Spain, covering about 97% of the region's population (5 million inhabitants). We created a cohort with all naïve patients with diagnosed AF [diagnosis code of International Classification of Diseases, Ninth Revision, Clinical Modification (ICD-9-CM) 427.31] who started treatment with oral anticoagulants (warfarin, acenocoumarol, dabigatran, rivaroxaban, apixaban) between November 2011 (date of the market launch of dabigatran) and February 2014, in each of the 24 Health Areas (HAs, the administrative and territorial management units) that make up the public health care provision network in the region. We defined the naïve population as those patients without anticoagulant treatment prescribed in the 12 months preceding the first prescription in our analysis time window. People without pharmaceutical/health coverage by VHA, mainly some government employees whose prescriptions are reimbursed by civil service insurers, and thus not included in the pharmacy databases of the VHA, and patients not registered in the municipal census (non-residents or temporary residents), or who left the region or who were disenrolled from VHA coverage for other causes, were excluded because of limitations on follow-up. A patient flowchart has already been published elsewhere in Frontiers (Rodríguez-Bernal et al., 2017).

### Source

Information was obtained from the electronic information systems of the VHA. The Population Information System (SIP) provides information on the population under VHA coverage and registers some demographic characteristics, including the geographical/contextual situation of each person and dates and causes of VHA discharge, including death. The Minimum Basic Dataset (MBDS) at hospital discharge is a synopsis of clinical and administrative information on all hospital discharges, including diagnoses and procedures. The electronic medical record for ambulatory care (EMR), available in all primary healthcare centers and ambulatory facilities, has information about diagnoses, personal, and family medical history, laboratory results, lifestyle, etc. as well as information about both physician prescriptions and dispensations from pharmacy claims. All the information in these systems is linked at an individual level through a unique identifier.

### Covariates

Variables potentially related to the risk of atrial fibrillation and the use of oral anticoagulants in the study population over the study period were considered. These included demographic characteristics, comorbidities, use of DOAC and healthcare resource utilization in the preceding 12 months. Based on comorbidity information, we calculated and added relevant patient-level risk predictor scores—CHADS2, CHA2DS2-VASC, and HAS-BLED scores– to the dataset.

### Analysis

We first described sociodemographic and clinical patient and healthcare utilization measures per Health Area. Second, to describe HA initiation patterns over time, we calculated monthly percentages of treatment initiation with either a VKA or a DOAC. Third, we examined the variability in the percentage of initiation with VKA and with each different DOAC per HA, based on data from the last trimester of the study window (Nov 2013 to Feb 2014). Fourth, to identify contextual and individual factors associated with differences in initiation patterns, we used multilevel regression analyses with random effects with patients (first level) nested within HAs (second level) and we compared the output of the empty model (a model considering only the HA component) with models adding individual variables (Model I) and risk scores (Models II and III). Finally, we ranked HAs with respect to the regional average of the percentage of initiation to visualize inter-area differences by means of a caterpillar plot. All analyses were performed using R 3.2.3 (R Foundation) statistical software.

### Ethics

The study protocol was approved by the regional Ethics Committee for Clinical Research of the General Directorate of Public Health and the Centre for Public Health Research. Patient informed consent was not required because datasets were extracted with anonymized identifiers according to Spanish laws on privacy (Act 15/1999) and patients' rights (Act 41/2002).

## Results

### Patient characteristics and healthcare utilization

During the study period a total of 21,879 patients initiated treatment with an oral anticoagulant in the 24 Health Areas. For the annual period from November 2011 to November 2012, the total ratio of initiation with a DOAC was 14.6%. For the whole period, initiation with a DOAC was 18%, with HA rates ranging from 4.7 to 27.8%, thus implying a six-fold difference among HAs. In the month of November 2013, 2 years after the first prescription of a DOAC, the regional initiation share was 25.4%, ranging from 3.8 to 57.1%. Mean patient age was 75 years old and 48% of patients were females, with hypertension being by far the most frequent comorbidity (79%) followed by diabetes (31%) and history of bleeding or predisposition to bleeding (23%). The average number of medications per patient was 9.89, ranging from 8.70 to 10.99. The average ambulatory cardiology visits was 0.47, with HAs ranging from 0.13 to 0.85—again showing a six-fold difference among HAs (Table 1).

**Table 1:** Demographics, clinical characteristics, and healthcare utilization of the cohort per Health Areas.

	**All**	**A**	**B**	**C**	**D**	**E**	**F**	**G**	**H**	**I**	**J**	**K**	**L**	**M**	**N**	**O**	**P**	**Q**	**R**	**S**	**T**	**U**	**V**	**W**	**X**
*N* (%)	21,879	483 (2.21)	1,215 (5.55)	848 (3.88)	716 (3.27)	1,287 (5.88)	1,270 (5.80)	867 (3.96)	367 (1.68)	1,770 (8.09)	1,464 (6.69)	1,130 (5.16)	752 (3.44)	852 (3.89)	887 (4.05)	729 (3.33)	719 (3.29)	961 (4.39)	789 (3.61)	1,061 (4.85)	651 (2.98)	672 (3.07)	1,024 (4.68)	770 (3.52)	595 (2.72)
Female (%)	48	45	45	50	48	50	47	52	44	49	53	48	47	43	49	50	40	45	48	49	47	51	38	47	50
Age (mean)	75	75.65	74.06	74.68	76.09	75.05	74.82	75.41	76.22	74.79	75.26	74.32	74.48	73.65	75.1	76.28	72	74.32	74.11	73.95	73.52	74.17	72.46	74.78	73.2
DOAC (%)	3,932 (17.97)	26.1	24.5	27.5	13.4	22.5	6.9	8.8	7.4	12.2	11.4	25.2	20.5	15.7	25.0	25.2	19.6	23.5	16.2	20.3	16.3	27.8	23.1	4.7	10.4
INCOME*, EUROS (%)																									
<18.000	83.7	88.4	78.0	87.2	79.8	77.5	85.4	81.4	90.5	81.4	77.6	89.4	87.4	84.8	90.5	88.6	80.1	71.8	88.6	82.8	86.4	89.1	83.5	89.6	92.9
18.000-100.000	16.0	11.4	21.7	12.4	20.2	21.7	14.3	18.5	9.5	18.4	22.2	10.5	12.6	14.8	9.4	10.7	19.4	27.6	11.2	17.1	13.1	10.6	16.3	10.3	7.1
>100.000	0.3	0.2	0.3	0.4	0.0	0.8	0.3	0.1	0.0	0.3	0.2	0.2	0.0	0.4	0.1	0.7	0.4	0.6	0.3	0.1	0.5	0.3	0.2	0.1	0.0
**COMORBIDITIES (%)**																									
Congestive heart failure	21	25	22	25	24	20	20	20	16	21	22	21	22	17	25	14	21	20	20	22	25	22	21	24	24
Hypertension	79	77	77	82	83	78	81	80	80	79	79	80	81	79	81	81	78	76	77	79	75	85	78	84	78
Diabetes	31	28	31	32	33	33	31	32	31	34	35	32	28	30	31	25	27	28	25	28	32	31	27	39	30
Liver disease	6	4	6	4	5	6	5	9	5	6	7	6	5	4	6	6	6	6	7	8	6	8	8	8	8
Renal disease	12	9	13	13	12	11	13	10	13	12	12	9	12	8	11	9	10	10	10	13	10	13	16	18	16
Dementia	7	6	6	7	5	7	7	7	5	8	6	5	9	4	12	8	4	6	6	7	7	7	7	7	8
Previous ischemic stroke or TIA	14	13	16	13	14	15	16	13	15	13	13	14	16	12	14	13	15	14	13	16	14	21	15	15	15
Coronary artery disease	21	14	19	21	22	19	23	20	25	21	20	20	24	23	25	17	21	21	26	19	18	20	23	24	20
Deep vein thromboembolism or pulmonary embolism	6	6	4	8	5	5	6	5	6	7	7	7	8	8	9	7	4	5	5	4	5	7	4	8	6
Hemorrhagic stroke	1	0	1	1	1	1	1	1	0	1	1	1	1	1	1	1	1	0	1	1	2	2	1	1	0
Gastrointestinal bleeding	4	4	2	4	4	4	3	3	4	4	4	3	5	3	3	3	4	3	5	5	4	5	3	3	3
Other major bleeding	21	22	17	21	29	20	21	24	23	23	21	16	20	17	26	18	18	19	21	23	20	23	14	25	19
Bleeding history or predisposition	23	23	19	23	32	23	23	26	25	26	24	19	23	19	29	19	21	21	24	26	23	25	16	27	21
CHADS2 score (mean)	2.17	2.16	2.20	2.24	2.34	2.22	2.21	2.18	2.22	2.17	2.22	2.19	2.21	2.02	2.24	2.10	2.03	2.08	2.07	2.15	2.11	2.34	2.00	2.35	2.13
CHA2DS2-VASC score (mean)	3.83	3.78	3.77	3.94	4.01	3.88	3.89	3.87	3.90	3.87	3.95	3.84	3.92	3.67	4.00	3.82	3.52	3.69	3.71	3.75	3.70	4.06	3.55	4.09	3.76
HAS-BLED score (mean)	2.21	2.15	2.17	2.23	2.34	2.20	2.26	2.27	2.26	2.22	2.24	2.14	2.23	2.08	2.28	2.20	2.14	2.12	2.15	2.26	2.13	2.42	2.22	2.41	2.19
**HEALTHCARE UTILIZATION (MEAN)**																									
Number of medications	9.89	8.70	9.30	10.17	10.22	10.26	9.94	10.06	10.28	10.36	10.15	10.16	10.44	9.01	10.95	9.80	8.85	9.32	9.69	9.76	9.40	10.15	8.79	10.99	9.87
Hospitalizations	0.66	0.55	0.67	0.64	0.62	0.59	0.74	0.55	0.56	0.48	0.67	0.62	0.69	0.59	0.66	0.57	0.70	0.72	0.81	0.67	0.89	1.10	0.70	0.73	0.72
Emergency department visits	1.22	1.37	1.25	1.32	1.55	1.29	1.48	1.72	1.35	1.29	1.43	0.52	0.88	1.11	1.36	0.65	1.28	0.69	1.60	0.24	1.50	1.70	1.15	1.60	1.48
**AMBULATORY VISITS (MEAN)**																									
Cardiologist visits	0.47	0.54	0.54	0.57	0.13	0.45	0.44	0.51	0.45	0.56	0.22	0.22	0.27	0.28	0.71	0.50	0.62	0.76	0.59	0.85	0.81	0.54	0.19	0.38	0.35
Neurologist visits	0.14	0.17	0.24	0.12	0.04	0.16	0.12	0.14	0.19	0.17	0.08	0.04	0.21	0.05	0.13	0.20	0.26	0.17	0.14	0.24	0.27	0.25	0.03	0.10	0.09
Social work vistis	0.09	0.08	0.11	0.15	0.13	0.07	0.07	0.07	0.07	0.09	0.06	0.05	0.06	0.05	0.05	0.24	0.16	0.12	0.06	0.17	0.10	0.02	0.02	0.09	0.13
Mental health visits	0.09	0.07	0.08	0.11	0.12	0.09	0.08	0.12	0.09	0.09	0.04	0.05	0.06	0.08	0.11	0.13	0.13	0.08	0.15	0.20	0.10	0.07	0.03	0.05	0.19
Hospitalization in 30 days before treatment initiation (%)	31	25	33	31	31	26	37	24	24	21	32	27	34	24	24	30	37	36	39	31	49	51	31	23	35

*Income information was only available for 20,850 patients; income information for the remaining 1,029 patients is missing*.

*Some information is presented in means (healthcare utilisation, risk scores, and ambulatory visits). Average numbers are calculated as simple means. For instance, in the case of ambulatory visits, the figures are calculated by dividing the total number of visits (to cardiologist, neurologist, social work or mental health) in the period by the total number of patients*.

### Initiation time trends

**Figure 1:**
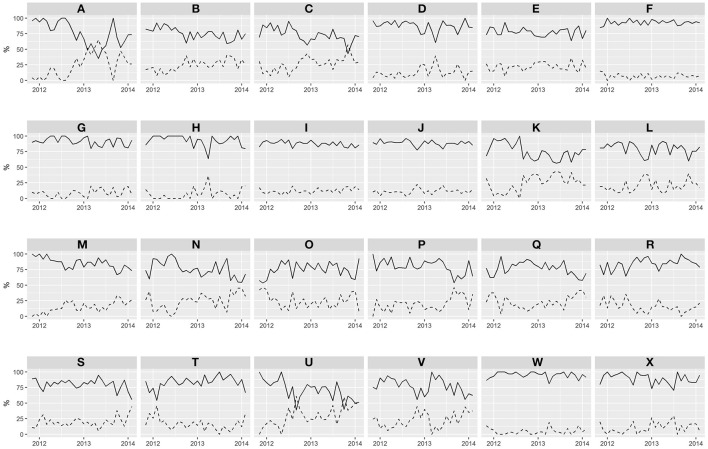
Time trends of the percentage of initiation with VKA or DOAC per Health Areas over time. Letters A to X represents the 24 HAs. Solid line: % of initiation with VKA. Dotted line: % of initiation with DOAC.

Descriptive time trends of relative–VKA vs. DOAC–treatment initiation in the 24 HAs are presented in Figure 1. Trends in DOAC uptake differ among HAs, with some areas maintaining a modest introduction of DOAC as an initial therapy throughout the study period (for instance, areas F, G, I, J, or W show a monthly DOAC initiation share of below 25% for the whole period), while others show increasing levels of DOAC penetration over time (see areas B, K, N, P, or Q, where DOAC initiation rises from 25 to 50% at the end of the study window). Still, in some HAs the pace of DOAC initiation is sharp enough to overtake the ratio of VKA initial prescription at specific moments of time (areas A, C, and U where % of DOAC initiation surpasses 50% in certain months).

**Figure 2:**
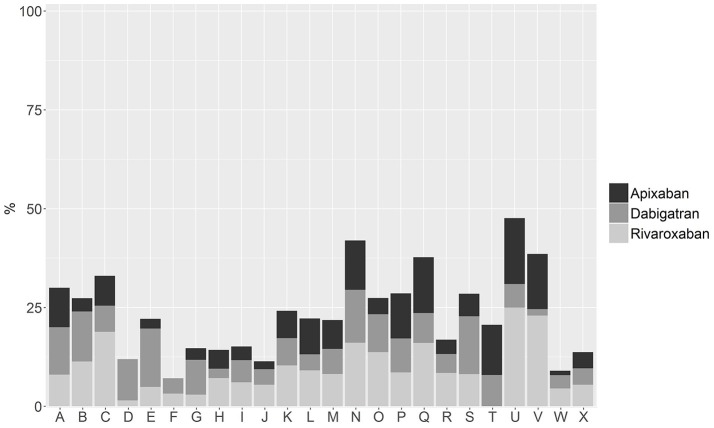
Choice of DOAC as initiation therapy per Health Area, expressed as a percentage of total initiation, for the period December 2013 to February 2014.

With regard to the choice of DOAC per HA, Figure 2 shows that the relative percentage of initiation with apixaban, dabigatran, or rivaroxaban in the last trimester of the study period (Dec 2013 to Feb 2014) is highly variable. For instance, in areas D and F apixaban is not prescribed while it leads area T with 1.7% of the initiation share. Rivaroxaban is not prescribed in Area T but is the leading DOAC of choice for initiation in 15 HAs, with a share of up to 25% of initiations in one area. The first *comer*, dabigatran, 2 years after its launch, still leads initiation in seven territories but with more modest shares, always below 15%. Also worth noting is the variation in the relative penetration of total DOAC initiation, with HAs ranging from 7.1% of initiation with DOAC for the considered trimester to 47.6%, thus entailing once again a more-than six-fold difference among areas.

### Analysis of associations

Table 2 presents a multilevel analysis showing the specific associations between individual and contextual (Health Area) characteristics and DOAC initiation in the study period as well as the analysis of variance and the values of the Intra-Class Correlation statistic (ICC) for the different models. Regarding the influence of the HA on initiation patterns, variability among HAs was statistically significant. ICC was 8.51% in the empty model, 8.07, 8.04, and 8.12% in models I, II, and III, respectively. Median Odds Ratio of the effect of HA heterogeneity on initiation variability was 1.70 for the empty model and 1.67 for models I, II, and III.

**Table 2:** Predicting factors of DOAC initiation. Multilevel analysis results.

**Fixed Effects**	**Empty Model**		**Model I**		**Model II**		**Model III**	
			**OR**	**95% CI**	**OR**	**95% CI**	**OR**	**95% CI**
Age, years			1.00	(0.99–1.00)	–	–	–	–
Sex			1.06	(0.98–1.14)	–	–	1.07	(0.99–1.16)
Income								
<18,000 €			Reference		Reference		Reference	
18,000–100,000 €			1.47	(1.34–1.62)	1.44	(1.31–1.59)	1.46	(1.33–1.60)
>100,000 €			2.50	(1.45–4.29)	2.43	(1.41–4.18)	2.49	(1.45–4.28)
**COMORBIDITY**								
Diabetes			0.87	(0.80–0.95)	–	–	0.87	(0.80–0.94)
Congestive heart failure			0.89	(0.81–0.98)	–	–	0.86	(0.78–0.95)
Previous ischemic stroke or TIA			1.31	(1.17–1.45)	–	–	–	–
Renal disease			0.68	(0.59–0.77)	0.66	(0.58–0.75)	–	–
Dementia			1.29	(1.12–1.50)	1.32	(1.15–1.53)	1.32	(1.14–1.52)
Coronary artery disease			0.91	(0.83–1.01)	–	–	0.91	(0.83–1.00)
Deep vein thromboembolism or pulmonary embolism			0.68	(0.57–0.81)	–	–	0.67	(0.56–0.79)
Hemorrhagic stroke			1.73	(1.21–2.46)	1.86	(1.31–2.64)	–	–
**CHADS2–VASC**								
Score 0			–	–	Reference		–	–
Score 1			–	–	0.71	(0.55–0.90)	–	–
Score ≥2			–	–	0.63	(0.51–0.77)	–	–
**HAS–BLED SCORE**								
Score 0			–	–	–	–	Reference	
Score 1			–	–	–	–	0.83	(0.69–1.00)
Score ≥2			–	–	–	–	0.80	(0.67–0.96)
**HEALTHCARE UTILIZATION**								
Number of medications (≥6)			0.91	(0.82–1.01)	0.96	(0.87–1.06)	0.95	(0.86–1.05)
Emergency Department visits (≥1)			0.85	(0.78–0.92)	0.84	(0.78–0.91)	0.86	(0.79–0.93)
Cardiologist visits (≥1)			1.93	(1.78–2.08)	1.85	(1.71–2.00)	1.88	(1.73–2.03)
Neurologist visits (≥1)			1.14	(1.00–1.30)	1.27	(1.12–1.43)	1.29	(1.13–1.46)
**Random Effects**								
Area intercept variance (*SD)*	0.3060 (0.553)		0.2889 (0.537)		0.2877 (0.536)		0.2909 (0.539)	
Median Odds Ratio	1.70		1.67		1.67		1.67	
Intraclass Correlation	8.51%		8.07%		8.04%		8.12%	
Area under the ROC curve (CI: 95%)	0.634 (0.624–0.643)		0.679 (0.670–0.688)		0.676 (0.667–0.685)		0.675 (0.665–0.684)	
AIC	19,952		18,636		18,678		18,696	

*CHA2DS2-VASC = Congestive heart failure, hypertension, age > 75, diabetes mellitus, stroke, vascular disease, age 65–64, sex category. HAS-BLED = Hypertension, abnormal renal/liver function, stroke, bleeding history, or predisposition, labile international normalized ratio, age > 65, drugs/alcohol concomitantly*.

In Model I, where individual characteristics are added to the empty model, DOAC-initiating patients were less likely to have comorbidities such as diabetes (OR:0.87), renal disease (OR:0.68), deep vein thromboembolism or pulmonary embolism (OR:0.68), but were more likely to present episodes of previous stroke or TIA (OR:1.31) and hemorrhagic stroke (OR:1.73) when compared with patients initiating with VKA treatment. Patients initiating with DOAC showed fewer ED (OR:0.85) visits but more visits to a cardiologist in the 12 months preceding the index prescription (OR:1.93). In Models II and III, in which we incorporate risk prediction scores, we found that an increased risk of stroke and bleeding as captured by CHADS2 and HAS BLED scores was significantly associated with lower odds of DOAC initiation. Also, higher income was associated with a significant increase in the likelihood of initiating with a DOAC instead of VKA (OR: 2.50, 2.43, and 2.49 in Models I, II, and III).

With regard to discriminatory accuracy, AUC in the empty model was 0.634. Information provided by individual variables in model I resulted in an increase of AUC over the empty model of 0.045 units. Inclusion of risk scores in models II and III did not further increase discriminatory ability.

**Figure 3:**
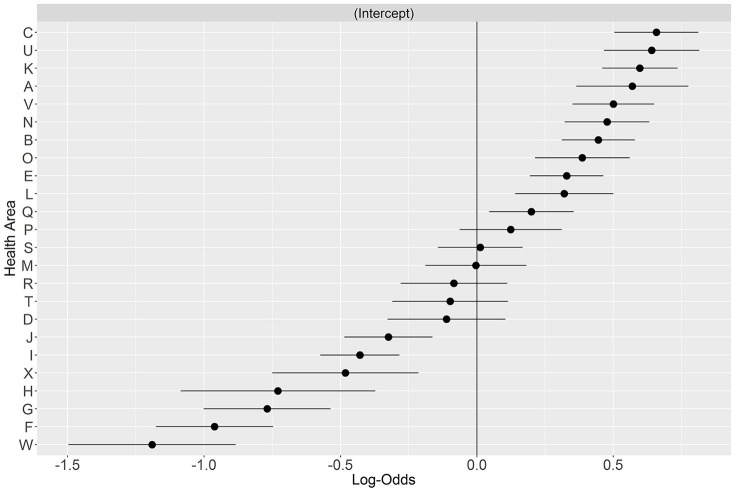
Variation in initiation with a DOAC per Health Area. HAs are ranked with respect to the regional average.

Finally, we ranked HAs according to their mean likelihood of DOAC initiation with regard to the regional average—see Figure 3. In seven HAs the propensity for DOAC prescription was significantly below the regional average, in 11 the propensity for DOAC prescription was significantly higher than average, and six areas showed no difference with the regional average.

## Discussion

We found that HAs are contextual level factors that significantly influence patterns of oral anticoagulation initiation in patients with atrial fibrillation in the region of Valencia, regardless of individual characteristics. The HA of residence was, together with some individual variables, one of the most important factors of variation in the choice of anticoagulant treatment initiation. At patient level, initiation with DOAC was associated with fewer concomitant comorbidities, lower risk of bleeding and stroke according to prediction indexes scores, presence of previous ischemic or hemorrhagic stroke, wealthier status, and with more visits to the cardiologist. To our knowledge, this is the first study to evaluate contextual and individual level factors associated with the initiation of oral anticoagulant therapy in a real world setting.

We observed a marked heterogeneity in the initiation trends among Health Areas, with a high variability in the percentage of DOAC prescription and in the choice of DOAC. Many factors, mainly supply side elements, may be associated with such different trends: inter medical-inspector variability in prior-authorization decision-making, level of ability of the HA pharmacy managers to influence specialist and primary care prescription, or different prescriber risk aversion in the face of uncertainties when prescribing. In some HAs DOAC initiation peaks coinciding in time with DOAC launches can be observed, suggesting that a variation gradient may come from differential promotion intensity and sensitivity. The variations observed in the patterns of oral anticoagulant initiation among patients with atrial fibrillation, and the ascertainment that the contextual factor “Health Area” exerts a noticeable influence on initiation patterns, may have important implications for patients, payers and the health care system.

The relatively low uptake of DOAC observed in our study when compared to other healthcare systems (Desai et al., 2014; Olesen et al., 2015) is consistent with the formal restriction of DOAC as a second line therapy for stroke prevention in atrial fibrillation patients. Also, renal failure patients were less likely to be initiated with DOAC, which is reasonable as renal precautions are stated on every DOAC Summary of Product Characteristics. Similarly, patients with a previous stroke (ischemic or hemorrhagic) were more likely to initiate on DOACs, which seems logical as those conditions are part of the prior authorization criteria for initiating treatment with a DOAC. On the other hand, some of our results are less foreseeable. Patients that initiate with a DOAC seem to be at lower risk, and this should be taken into account when considering real-life effectiveness. Exploring whether health outcomes are affected by anticoagulant choice (and by DOAC choice when a DOAC is prescribed) and at what cost, should be a research priority to gain a better understanding of the implications of initiation variability and ultimately to be able to provide policy guidance on the matter. Finally, we found that higher income is strongly associated with more than a two-fold odds of being initiated with a DOAC. This could be explained by cost-sharing considerations (González López-Valcárcel et al., 2017), as DOAC entails a higher cost burden to patients than VKA, and doctors may be acting as financial (in addition to clinical) agents to patients (González López-Valcárcel et al., 2011) nevertheless it is a disturbing result that causes concern with regard to the universal and equal access to healthcare resources in the Spanish National Health System.

## Limitations

Our study is subject to some limitations. We considered the simplest possible multilevel structure of individuals nested within Health Areas, but we acknowledge that other contexts and/or care levels may influence prescription—such as primary care areas or physicians. However, this is the most common design in neighborhood and health studies (Merlo et al., 2006), and the variables we included in our analyses are appropriate to evaluate differences in the initiation of oral anticoagulation. Despite including many relevant individual variables in our analysis, we cannot rule out the existence of omitted confounding factors; the relatively low values of AUC evidence that the factors considered had a modest discriminatory capacity to distinguish between VKA and DOAC initiators. For instance, we could not include information on the impossibility of accessing INR control facilities or regarding the presence of a contraindication to VKA, as these data are not routinely recorded in linkable clinical databases. These factors could explain some contextual and individual heterogeneity and further research should examine their influence on initiation variation, but their absence does not affect the relevance of our results. Still, in multilevel analyses it is always critical to distinguish between confounder and mediator factors given that place of residence, as well as clinical outcomes, may act as mediators of the Health Area effect (Merlo et al., 2006). Furthermore, cautiousness is needed when establishing causal relationships with observational designs, and neighborhood and health studies are not an exception (Diez Roux, 2004; Merlo et al., 2016). Some contextual effects may be determined by the individual composition of the Health Area while others may be due to independent factors (e.g., better access to a specialist cardiology unit). All in all, our results should be interpreted with caution, and transferability to other contexts may be limited to the Spanish territory.

## Conclusions

The main finding of this study is that initiation with oral anticoagulants was influenced by factors both at the patient and the HA level, with the HA level having a greater impact on initiation behavior than many individual variables. The fact that factors not directly related to clinical appropriateness are influencing treatment initiation choice exposes important management issues. Among those, and according to our results, interventions aimed at improving DOAC initiation decision making and at reducing variability should take into account the Health Area component.

In Spain, prior authorization criteria relegate DOAC to a second line therapy. Based on this assumption, low rates of DOAC initiation throughout the country would be expected. However, in our study we showed that in some Health Areas roughly 50% of patients initiate with a DOAC in the region of Valencia, and visiting a cardiologist is associated with a relatively higher initiation with a DOAC. Within Health Areas, the choice of DOAC was also very variable, raising the need to generate more evidence about the factors affecting anticoagulant initiation decision making. Also, the linkage of utilization studies with real-world comparative safety and effectiveness analyses is needed to better seize the pros and cons—and the why's—of new oral anticoagulant use in daily clinical practice as a way to take full advantage of their potential benefits.

## Author contributions

GS, SP, and AG had full access to all the data in the study and take responsibility for its integrity and the accuracy of its analysis. The study was designed by SP and GS and carried out by AG, DB, JL, CR, SP, and GS. JL and DB carried out the data preparation and the statistical analysis. AG drafted the manuscript. All authors participated in the analysis and interpretation of data and critical revision of the manuscript for important intellectual content. All approved the final version submitted for publication and agree to be accountable for all aspects of the work by ensuring that questions related to the accuracy or integrity of any part of the work are appropriately investigated and resolved.

### Conflict of interest statement

AG is a former employee of Boehringer-Ingelheim. GS has participated in an advisory board meeting for Boehringer-Ingelheim, and SP has participated in scientific meetings for Novartis and Ferrer International. The other authors declare that the research was conducted in the absence of any commercial or financial relationships that could be construed as a potential conflict of interest.

## References

[B1] (1991). Stroke prevention in atrial fibrillation study. Final Results.. Circulation 84, 527–539. 10.1161/01.CIR.84.2.527 1860198

[B2] (1994). Warfarin versus aspirin for prevention of thromboembolism in atrial fibrillation: stroke prevention in atrial fibrillation ii study. Lancet 343, 687–691 7907677

[B3] ACOD (2016). INFORME DE POSICIONAMIENTO TERAPÉUTICO UT_ACOD/V5/21112016. Criterios Y Recomendaciones Generales Para El Uso De Los Anticoagulantes Orales Directos (ACOD) En La Prevención Del Ictus Y La Embolia Sistémica En Pacientes Con Fibrilación Auricular No Valvular. Ministerio de Sanidad, Servicios Sociales e Igualdad Madrid Available online at: https://www.aemps.gob.es/medicamentosUsoHumano/informesPublicos/docs/criterios-anticoagulantes-orales.pdf).

[B4] Anis A. H. Carruthers G. Carter A. O. Kierulf J. (1996). Variability in prescription drug utilization: issues for research. Can. Med. Assoc. J. 154, 635–640 8603319 PMC1487542

[B5] Brais C. Larochelle J. Turgeon M. Tousignant A. Blais L. Perreault S.. (2015). Patterns of oral anticoagulants use in atrial fibrillation. J. Popul. Ther. Clin. Pharmacol. 22:e90–e95. 25715385

[B6] Chugh S. S. Havmoeller R. Narayanan K. Singh D. Rienstra M. Benjamin E. J.. (2014). Worldwide epidemiology of atrial fibrillation: a global burden of disease 2010 study. Circulation 129, 837–847. 10.1161/CIRCULATIONAHA.113.005119 24345399 PMC4151302

[B7] Connolly S. J. Ezekowitz M. D. Yusuf S. Eikelboom J. Oldgren J. Parekh A.. (2009). Dabigatran versus warfarin in patients with atrial fibrillation. N. Engl. J. Med. 361, 1139–1151. 10.1056/NEJMoa0905561 19717844

[B8] Desai N. R. Krumme A. A. Schneeweiss S. Shrank W. H. Brill G. Pezalla E. J.. (2014). Patterns of initiation of oral anticoagulants in patients with atrial fibrillation- quality and cost implications. Am. J. Med. 127, 1075–1082. 10.1016/j.amjmed.2014.05.013 24859719

[B9] Diez Roux A. V. (2004). The study of group-level factors in epidemiology: rethinking variables, study designs, and analytical approaches. Epidemiol. Rev. 26, 104–111. 10.1093/epirev/mxh006 15234951

[B10] Donohue J. M. Morden N. E. Gellad W. F. Bynum J. P. Zhou W. Hanlon J. T.. (2012). Sources of regional variation in medicare part D drug spending. N. Engl. J. Med. 366, 530–538. 10.1056/NEJMsa1104816 22316446 PMC3285245

[B11] Dubois R. W. Batchlor E. Wade S. (2002). Geographic variation in the use of medications: is uniformity good news or bad? Health Aff. 21, 240–250. 10.1377/hlthaff.21.1.240 11900083

[B12] Eisenberg J. M. (2002). Physician utilization: the state of research about physicians' practice patterns. Med. Care 40, 1016–1035 10.1097/00005650-200211000-00004 12409848

[B13] Fisher E. S. Wennberg D. E. Stukel T. A. Gottlieb D. J. Lucas F. L. Pinder E. L. (2003a). The implications of regional variations in medicare spending. Part 1: the content, quality, and accessibility of care. Ann. Intern. Med. 138, 273–287. 10.7326/0003-4819-138-4-200302180-00006 12585825

[B14] Fisher E. S. Wennberg D. E. Stukel T. A. Gottlieb D. J. Lucas F. L. Pinder E. L. (2003b). The implications of regional variations in Medicare spending. Part 2: health outcomes and satisfaction with care. Ann. Intern. Med. 138, 288–298. 10.7326/0003-4819-138-4-200302180-00007 12585826

[B15] Fuster V. Bhatt D. L. Califf R. M. Michelson A. D. Sabatine M. S. Angiolillo D. J.. (2012). Guided antithrombotic therapy: current status and future research direction: report on a national heart, lung and blood institute working group. Circulation 126, 1645–1662. 10.1161/CIRCULATIONAHA.112.105908 23008471 PMC4086864

[B16] Giugliano R. P. Ruff C. T. Braunwald E. Murphy S. A. Wiviott S. D. Halperin J. L.. (2013). Edoxaban versus warfarin in patients with atrial fibrillation. N. Engl. J. Med. 369, 2093–2104. 10.1056/NEJMoa1310907 24251359

[B17] González López-Valcárcel B. Librero J. García-Sempere A. Peña L. M. Bauer S. Puig-Junoy J. Peiró S.. (2017). Effect of cost sharing on adherence to evidence-based medications in patients with acute coronary syndrome. Heart 103, 1082–1088. 10.1136/heartjnl-2016-310610 28249992 PMC5566093

[B18] González López-Valcárcel B. Librero J. Sanfélix-Gimeno G. Peiró S. Group for Drug Utilization Research in the Spanish National Health System (IUM-SNS Group) (2011). Are prescribing doctors sensitive to the price that their patients have to pay in the Spanish National Health System? BMC Health Serv. Res. 11:333. 10.1186/1472-6963-11-333 22151628 PMC3265431

[B19] Granger C. B. Alexander J. H. McMurray J. J. Lopes R. D. Hylek E. M. Hanna M.. (2011). Apixaban versus warfarin in patients with atrial fibrillation. N. Engl. J. Med. 365, 981–992. 10.1056/NEJMoa1107039 21870978

[B20] January C. T. Wann L. S. Alpert J. S. Calkins H. Cigarroa J. E. Cleveland J. C. Jr.. (2014). AHA/ACC/HRS guideline for the management of patients with atrial fibrillation: executive summary: a report of the American College of Cardiology/American Heart Association Task Force on practice guidelines and the heart rhythm society. Circulation 130, 2071–2104. 10.1161/CIR.0000000000000040 24682348

[B21] Jørgensen H. S. Nakayama H. Reith J. Raaschou H. O. Olsen T. S. (1996). Acute stroke with atrial fibrillation. The copenhagen stroke study. Stroke 27, 1765–1769. 10.1161/01.STR.27.10.1765 8841326

[B22] Lancaster T. R. Singer D. E. Sheehan M. A. Oertel L. B. Maraventano S. W. Hughes R. A.. (1991). The impact of long-term warfarin therapy on quality of life. Evidence from a randomized trial. Boston area anticoagulation trial for atrial fibrillation investigators. Arch. Intern. Med. 151, 1944–1949. 10.1001/archinte.1991.00400100032005 1929681

[B23] Lin H. J. Wolf P. A. Kelly-Hayes M. Beiser A. S. Kase C. S. Benjamin E. J.. (1996). Stroke severity in atrial fibrillation. The framingham study. Stroke 27, 1760–1764. 10.1161/01.STR.27.10.1760 8841325

[B24] Merlo J. Chaix B. Ohlsson H. Beckman A. Johnell K. Hjerpe P.. (2006). A brief conceptual tutorial of multilevel analysis in social epidemiology: using measures of clustering in multilevel logistic regression to investigate contextual phenomena. J. Epidemiol. Community Health 60, 290–297. 10.1136/jech.2004.029454 16537344 PMC2566165

[B25] Merlo J. Wagner P. Ghith N. Leckie G. (2016). An original stepwise multilevel logistic regression analysis of discriminatory accuracy: the case of neighbourhoods and health. PLoS ONE 11:e0153778. 10.1371/journal.pone.0153778 27120054 PMC4847925

[B26] National Research Council (2013). Variation in Health Care Spending: Target Decision Making, Not Geography. (Washington, DC: The National Academies Press). 24851301

[B27] Olesen J. B. Sørensen R. Hansen M. L. Lamberts M. Weeke P. Mikkelsen A. P.. (2015). Non-vitamin K antagonist oral anticoagulation agents in anticoagulant naïve atrial fibrillation patients: Danish nationwide descriptive data 2011-2013. Europace 17, 187–193. 10.1093/europace/euu225 25236181

[B28] Patel M. R. Mahaffey K. W. Garg J. Pan G. Singer D. E. Hacke W.. (2011). Rivaroxaban versus warfarin in nonvalvular atrial fibrillation. N. Engl. J. Med. 365, 883–891. 10.1056/NEJMoa1009638 21830957

[B29] Patel P. A. Zhao X. Fonarow G. C. Lytle B. L. Smith E. E. Xian Y. (2015). Novel oral anticoagulant use among patients with atrial fibrillation hospitalized with ischemic stroke or transient ischemic attack. Circ. Cardiovasc. Qual. Outcomes 8, 383–392. 10.1161/CIRCOUTCOMES.114.000907 26058721 PMC4512906

[B30] Petersen P. Boysen G. Godtfredsen J. Andersen E. D. Andersen B. (1989). Placebo-controlled, randomised trial of warfarin and aspirin for prevention of thromboembolic complications in chronic atrial fibrillation. The copenhagen AFASAK study. Lancet 1, 175–179. 10.1016/S0140-6736(89)91200-2 2563096

[B31] Potpara T. S. Lip G. (2017). Postapproval observational studies of non–vitamin k antagonist oral anticoagulants in atrial fibrillation. JAMA 317, 1115–1116. 10.1001/jama.2017.1152 28208176

[B32] Rodríguez-Bernal C. L. Hurtado I. García-Sempere A. Peiró S. Sanfélix-Gimeno G. (2017). Oral anticoagulants initiation in patients with atrial fibrillation: real-world data from a population-based cohort. Front. Pharmacol. 8:63. 10.3389/fphar.2017.00063 28261098 PMC5314137

[B33] The Boston Area Anticoagulation Trial for Atrial Fibrillation Investigators Singer D. E. Hughes R. A. Gress D. R. Sheehan M. A. Oertel L. B.. (1990). The effect of low-dose warfarin on the risk of stroke in patients with nonrheumatic atrial fibrillation. N. Engl. J. Med. 323, 1505–1511. 10.1056/NEJM199011293232201 2233931

[B34] Wennberg J. E. Fisher E. S. Skinner J. S. (2002). Geography and the debate over medicare reform. Health Aff. 21, W96–W114. 10.1377/hlthaff.w2.96 12703563

[B35] Zhang Y. Baicker K. Newhouse J. P. (2010a). Geographic variation in medicare drug spending. N. Engl. J. Med. 363, 405–409. 10.1056/NEJMp1004872 20538621 PMC3364516

[B36] Zhang Y. Baicker K. Newhouse J. P. (2010b). Geographic variation in the quality of prescribing. N. Engl. J. Med. 363, 1985–1988. 10.1056/NEJMp1010220 21047217 PMC3047447

[B37] Zhang Y. Baik S. H. Fendrick A. M. Baicker K. (2012). Comparing local and regional variation in health care spending. N. Engl. J. Med. 367, 1724–1731. 10.1056/NEJMsa1203980 23113483 PMC3490218

